# Impact of training for site staff recruiting into trials of rare skin diseases: experiences from stop gap and blister

**DOI:** 10.1186/1745-6215-14-S1-P134

**Published:** 2013-11-29

**Authors:** Eleanor Mitchell, Lelia Duley, Hywel Williams, Diane Whitham

**Affiliations:** 1 University of Nottingham, Nottingham, UK

## Background

Nottingham Clinical Trials Unit is co-ordinating two RCTs in rare skin diseases: one recruited people with pyoderma gangrenosum (STOP GAP), the other people with bullous pemphigoid (BLISTER). Sites were initially identified via the UK Dermatology Clinical Trials Network (http://www.ukdctn.org) without using formal site selection criteria. Many sites were naive, having not recruited to trials previously.

Training of site staff used a variety of methods including investigator meetings, face-to-face site initiation meetings, telephone training and remote training using a DVD. This presentation will describe recruitment and data quality by trial site, and how these relate to the type of training.

## Methods

Training was assessed based on initial training, rather than training of new staff who joined the trial later. Recruitment by site and trends in recruitment were compared for sites having different forms of training. Completeness of data collection, number of data queries, and timeliness of returns for data and queries were also compared.

## Results

Across the 2 trials, there were 67 UK sites. STOP GAP recruited 121 participants over 3½ years. BLISTER aims to recruit 256 participants by the time recruitment closes in September 2013. Results will be presented and discussed.

## Conclusion

Recruitment and data quality are key elements of efficient trial conduct. Results from this study will contribute to understanding of how training of site staff might improve study conduct for trials involving naive sites and those involving participants with rare diseases.

